# Seeing the Forest Through the Trees

**DOI:** 10.1016/j.jacadv.2025.101655

**Published:** 2025-03-16

**Authors:** Jennifer N. Avari Silva, Anthony G. Pompa

**Affiliations:** a Department of Pediatrics, Washington University in St. Louis School of Medicine, St. Louis, Missouri, USA; b Department of Biomedical Engineering, McKelvey School of Engineering, Washington University in St. Louis, St. Louis, Missouri, USA

**Keywords:** AI, ICM, remote patient monitoring (RPM)

**Figure:**
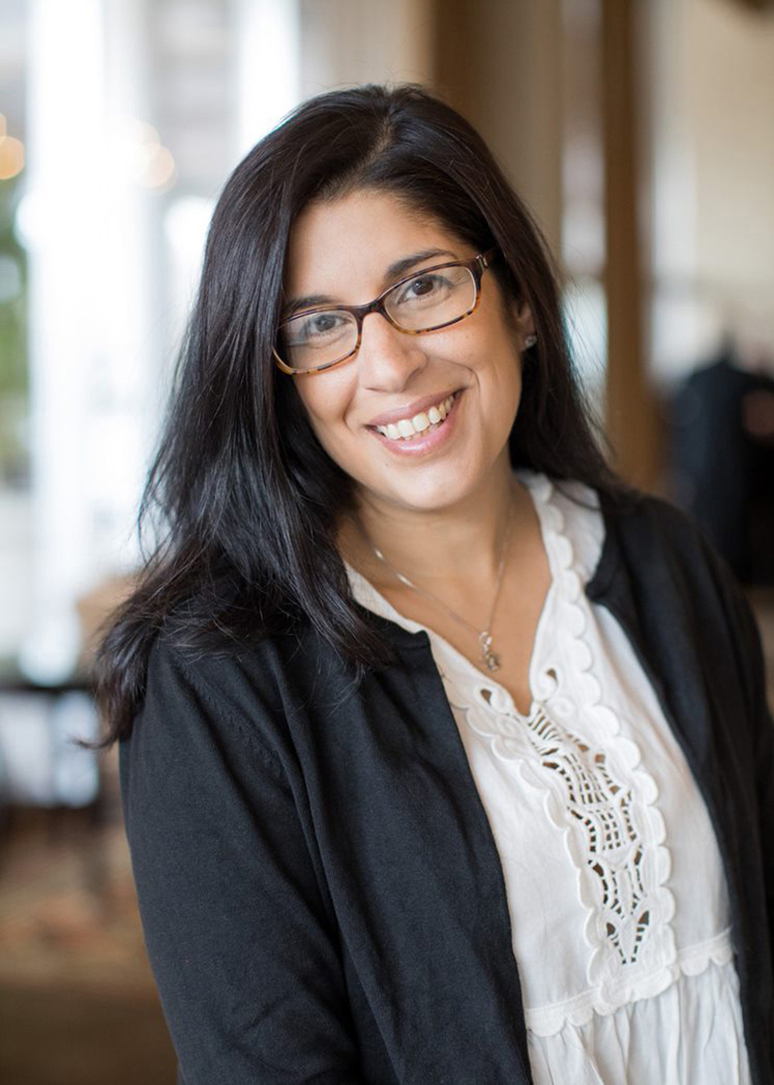

“Our life is frittered away by detail. Simplify, simplify, simplify!”

—Henry David Thoreau

In this issue of *JACC: Advances*, Katapadi et al^1^ present their work entitled “Impact of Artificial Intelligence-Enhanced Insertable Cardiac Monitors on Device Clinic Workflow and Resource Utilization.” The authors evaluated the potential impact of insertable cardiac monitors (ICMs) with embedded artificial intelligence (AI) algorithms on the clinical workflow and financial sustainability of remote patient monitoring (RPM) clinics by performing a cross-sectional analysis of 140 device clinics between July 2022 and April 2024. To assess this, deidentified data from over 19,000 remote monitoring patients with AI-enhanced and non-AI-enhanced ICMs were used to extrapolate the effect that a medium to large size RPM clinic may expect from manufacturer specific AI algorithm. Specific outcome measures included assessment of how AI-enabled ICMs impacted the burden of nonactionable alerts (NAAs) and clinic staffing requirements based on current guidelines.

The model demonstrated that AI-enhanced ICMs resulted in a 58.5% annual reduction in NAAs, resulting in a staffing cost savings of 559 hours annually. As such, it was estimated that a center following 600 ICMs would have a decrease in annual staffing costs of $29,470 when AI-enhanced ICM algorithms were employed. The conclusion was that AI-enhanced ICMs could significantly lower the burden of NAAs and thereby lead to a reduction in device clinic workloads and staffing costs.

Over the past several years, there has been a significant increase in the number of ICM implantations, increasing from 5 procedures/million enrollees in 2013 to 11 procedures/million enrollees between 2015 and 2018^2^ for a variety of reasons including technology miniaturization, ease of implantation (including a shift to outpatient procedures), and growth of remote monitoring. Guidelines for both adult and pediatric patients have reflected this change in practice with expanded indications, including monitoring atrial fibrillation recurrence, occult atrial fibrillation burden, unexplained syncope, and management guidance for patients with genetic arrhythmia syndromes.^3^^,^^4^ The maximal utility of these devices is achieved when they are coupled with a remote monitoring clinic.

The Heart Rhythm Society has crafted guidelines for proper staffing models for remote monitoring clinics as well as appropriate use of remote monitoring.^5^ For all patients with an implantable cardiac device, remote monitoring remains the standard of care. However, as the indications for ICMs expand, so too does the remote monitoring burden. Inherently, this has a downstream result of increasing NAAs. These alerts can consume valuable clinic staff time, reducing resources that could otherwise be allocated toward evaluating and managing actionable alerts.

AI has the potential to dramatically reduce the burden of NAAs and improve the remote monitoring experience for patients and staff. This study evaluates the utility of edge AI, defined as the use of AI algorithms and models on local devices, and allows for real-time data processing and analysis without relying on the cloud^6^ or connectivity (see Figure 1). Edge AI can be a powerful, albeit limited, tool in that edge AI has low latency with strong security measures, though restricted computational power. This contrasts with cloud-based AI strategies, which can aggregate large volumes of data and have significantly greater computational power but do introduce latency into the feedback system. In cardiac RPM, there has been an exponential increase in the number of cloud-based AI systems in place (such as Octagos, mentioned in the Katapadi et al^1^ paper), which adds an additional layer of cloud-based AI on top of the ICM’s edge AI. Future studies should evaluate the relative improvements that these layered AI systems (edge-based AI at the level of the ICM + cloud-based AI at the level of data aggregation and analysis systems) provide over edge AI alone or cloud-based AI alone. We predict that a layered AI approach will likely be the clinically relevant path forward, but more investigation is needed to determine the balance.

**Figure 1:**
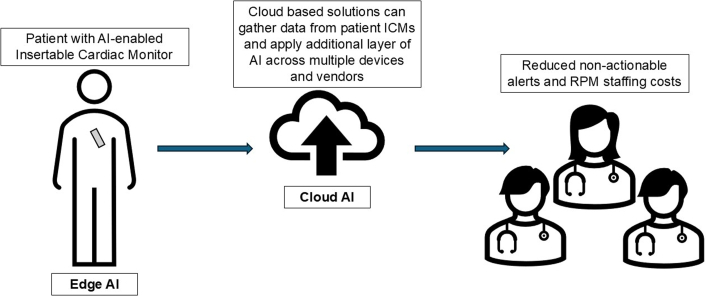
Flow of Data for Patients With Insertable Cardiac Monitors (ICMs), Including Where the Impact of Edge and Cloud-Based AI Occur

Additionally, our field has experienced significant reductions in reimbursement across electrophysiology services over the recent years, and a financially stable RPM program will be vital to the sustainability of outpatient electrophysiology practices. One can infer that the deluge of NAAs and volume of transmissions may not be adequately capturing all potential revenue. AI should be able to facilitate this process, making appropriate billing more efficient and sustainable, balancing human capabilities with layered AI.

However, the cost of innovation remains high. We would expect AI-enabled ICMs to have an increased upfront cost for hardware, while the backend cost of staffing these clinics will hopefully decrease over time. The benefits of AI-enhanced ICMs could make a substantial impact in any practice, including small, academic, and pediatric sites. Over time, this initial cost of innovation will be balanced out by creating financially sustainable RPM clinics and improving patient outcomes via improved rhythm surveillance.

Electrophysiologists and those staffing remote monitoring clinics should be encouraged by these data. AI-enhanced ICMs (edge AI) coupled with cloud-based AI solutions are here and can be employed to improve the resource utilization of our clinics. As improvements are made in these technologies and our understanding increases, we can dramatically decrease NAAs and create sustainable RPM clinics. The simplification of what needs to be reviewed by the staff can translate to more time directed toward developing plans for actionable alerts and spending more time with our patients instead of drowning in an inbox of artifacts.

## Funding support and author disclosures

Dr Silva serves as a consultant to iRhythm, Medtronic and Abbott; and has IP that has been licensed from Washington University to Sentiar and Excera. Dr Pompa has reported that he has no relationships relevant to the contents of this paper to disclose.
